# The Connections of Pregnancy-, Delivery-, and Infant-Related Risk Factors and Negative Life Events on Postpartum Depression and Their Role in First and Recurrent Depression

**DOI:** 10.1155/2016/2514317

**Published:** 2016-10-26

**Authors:** Pirjo Kettunen, Eeva Koistinen, Jukka Hintikka

**Affiliations:** ^1^Department of General Hospital Psychiatry, North Karelia Central Hospital, 80210 Joensuu, Finland; ^2^Department of Obstetrics and Gynecology, North Karelia Central Hospital, 80210 Joensuu, Finland; ^3^School of Medicine, University of Tampere, 33014 Tampere, Finland; ^4^Department of Psychiatry, Päijät-Häme Central Hospital, 15850 Lahti, Finland

## Abstract

*Introduction*. The aim of this study is to assess how negative life events and adverse experiences with pregnancy, delivery, the infant(s), and breastfeeding cessation impact on postpartum depression (PPD), specifically in first lifetime and recurrent depression.* Method*. The study group comprised 104 mothers with a current episode of PPD and a control group of 104 mothers who did not have current PPD. The Structured Clinical Interview for DSM-IV Axis I Disorders (SCID-I) was used for data collection. The course of the depression, adverse experiences, and breastfeeding were assessed by self-reports.* Results*. In age-adjusted multivariate analyses, mental and physical problems during pregnancy or delivery, postpartum problems with the infant and breastfeeding cessation, and negative life events during the previous 12 months were associated with postpartum depression. Eighteen percent (18%) of the mothers had first depression and 82% recurrent depression. Mental and physical problems during pregnancy or delivery were associated with both first lifetime and recurrent depression. Nevertheless, negative life events and infant/breastfeeding issues associated only with recurrent depression.* Conclusion*. Factors associated with pregnancy and delivery have an impact on PPD, but in recurrent depression other postnatal and psychosocial factors are also important risk factors.

## 1. Introduction

The postpartum period is unique with respect to the mother's psychosocial adjustment and the degree of physical changes. For most women, these experiences are constructive and mothers are healthy. Depression after childbirth, however, is common. The prevalence of postpartum depression (PPD) is 10–15% [1, 2]. PPD is usually associated with a previous history of depression also in primiparous women, but a subgroup of women may suffer depression only during the postpartum period [3–5]. There is some evidence that the occurrence of the first lifetime episode of depression during the postpartum period is associated with adverse events during the pregnancy or delivery, or with the infant [3]. Recurrent depressive episodes may have more predisposing factors during pregnancy and also psychosocial risk factors [3]. Stressful life events, adverse experiences before pregnancy, stress during pregnancy and delivery, and strain in infant care in general are connected to postpartum depression [2, 6–8]. Pregnancy and childbirth are often regarded as stressful life events in their own right [8], and childbirth procedures may act as a trigger for previous painful memories. Posttraumatic stress and depression after childbirth seem to be positively associated [9] and share the same underlying vulnerability factors, such as fear of childbirth, stress, and psychological problems [9].

Unplanned or unwanted pregnancy is a predictor of PPD [2, 7, 8]. An unexpected pregnancy may change the mother's life extensively and have social and economic implications; furthermore, difficulties in adjustment to parenthood may be greater if the pregnancy is not planned [7, 10, 11].

Depression and anxiety during pregnancy are significant contributors to PPD [2, 6–9]. Fear of childbirth [3, 9], feelings of stress [9, 12], and other psychological disorders [6] during pregnancy are also connected to PPD.

Pregnancy and delivery-related (obstetric) complications also cause physical and mental troubles for the mother and comprise a single predictor variable for PPD [2, 6, 8]. Obstetric factors, including pregnancy-related complications such as hyperemesis, preeclampsia, premature contractions and labor, hypertension, headache, pain, anemia, gestational diabetes, diabetes mellitus, and amniocentesis [3, 8, 12–15], as well as delivery-related complications, such as difficult and painful labor, cesarean section, instrumental delivery, premature delivery, and complicated puerperium-like excessive bleeding, have been examined as potential risk factors for PPD [2, 3, 8, 11]. In a recent Finnish study, cesarean section was a strong predisposing factor for PPD, specifically among those who had first lifetime depression [3]. The same study found that preterm birth associated with first lifetime depression, and anemia and gestational diabetes associated with recurrent depression in the postpartum period [3]. According to several studies, hyperemesis is linked with an increased risk for depression, anxiety, and mental health difficulties [16]. Infertility treatment does not increase the risk for PPD [3, 17].

The association between cesarean section and PPD is not simple. It is unclear whether delivery complications or long and painful labors leading to emergency procedures account for the association [8, 18]. Women who wanted vaginal delivery but delivered by cesarean section may be at an increased risk for PPD [19]. Conversely, women who had been diagnosed with a depressive disorder at the time of delivery have been shown to be significantly more likely to have a delivery by cesarean section [20].

Infant-related problems are often very stressful experiences for the mother. Mothers of premature infants, mothers of infants with illnesses/disabilities/distress, mothers of infants that are temperamentally difficult, and mothers who experience strain in childcare and have a lack of childcare knowledge are at risk for developing PPD [2, 3, 7, 15]. The relationship between problems with the infant and the mother's depression is likely to be complex; for example, the interrelationship between preterm birth and PPD may be explained by an interaction of multiple alterations in the labor and delivery processes, poorer-than-expected infant health outcomes, early parental stress, and dysfunctional mother-infant interaction [21, 22]. Specifically, infantile colic as a cause of excessive and prolonged crying is a well-known risk factor for depression [23, 24]. The amount of infant crying is associated with the experience of tiredness and fatigue in new mothers. Incremental exhaustion may trigger depressive symptoms, diminish the mother's ability to concentrate, and burden mother-child interaction [24].

Women who do not or fail to initiate breastfeeding are at risk for developing PPD [2, 10, 11, 25, 26]. The effect of breastfeeding on depression may be mediated by intentions to breastfeed. The risk for PPD may be higher among women who had planned to breastfeed and failed to do so [27]. The relationship between breastfeeding and PPD seems to be reciprocal. Breastfeeding reduces the risk for developing PPD and conversely PPD may decrease the rate of breastfeeding [10, 26, 28].

The relationship between stressful life events and postpartum depression is well known [2, 6, 8]. A number of life events and stressors are connected to PPD [11, 17, 29–31]. The mother's own sickness, the sicknesses or death of a significant other, problematic relationships, socioeconomic problems, and other traumatic experiences seem to be associated with depressive symptoms during the postpartum period [30, 31].

The postpartum period seems to be a time of vulnerability to depression. Ante- and postnatal periods present unique challenges in detecting the risk factors for PPD. The onset of depression may be unexpected, and it is useful to know risk factors for prevention and treatment. Little is known about the possible differences in risk factors for first lifetime and recurrent depression after childbirth. The aim of this study is to assess how pregnancy and delivery issues, issues relating to the infant and breastfeeding cessation, and negative life events associate with postpartum depression. In addition, the study seeks to discover if there are differences in their role between first lifetime depression and recurrent depression during the postpartum period.

## 2. Materials and Methods

The nature of this study is cross-sectional. Both the study group (depressed) and the control group (nondepressed) consisted of 104 mothers evaluated six weeks to six months after delivery. Mothers were recruited by primary health care nurses at antenatal clinics in Joensuu, a town in Eastern Finland, using the Edinburgh Postnatal Depression Scale (EPDS, range: 0–30) [32]. If the EPDS score was ≥10 or there was clinical suspicion of depression, the nurses told the mother that she could be assessed by a psychiatrist (Pirjo Kettunen) at the local General Hospital Psychiatric Unit at North Karelia Central Hospital, Joensuu, Finland. This community-based hospital unit serves a socioeconomically diverse population. Mothers were cared for at the Obstetric Department at the same hospital during pregnancy and delivery (approximately 1,550 deliveries per year). Data collection took place from 2003 to 2013. If the EPDS score was <10 and there was no clinical suspicion of depression, primary health care nurses asked mothers if they were willing to participate in the study as part of the control group and then organized a psychiatric assessment at the antenatal clinics. The control group was collected between 2008 and 2010.

The diagnoses of major depressive disorder in the study group (depressed mothers) and the control group (nondepressed mothers) were assessed by a psychiatrist (Pirjo Kettunen) by means of the Structured Clinical Interview for DSM-IV (*Diagnostic and Statistical Manual of Mental Disorders*, fourth edition) Axis I Disorders (SCID-I) [33]. Attendees with psychotic, addictive, and thyroid disorders were excluded. Women aged 18–40 years were included. The mothers were also evaluated by the psychiatrist (Pirjo Kettunen) using a semistructured interview designed to identify risk factors relating to pregnancy, delivery, the infant, breastfeeding, and life events during the previous year.

  Previous depressive episodes were assessed by asking whether the mothers had had depression lasting for at least two weeks; the time period was in line with DSM-IV [33]. Mothers were also asked how old they were and how many children, gestations, deliveries, spontaneous or induced abortions, and stillbirths they had had.

Mothers were asked about possible risk factors:

- *If the pregnancy was wanted or unwanted or if the mother could not say.* Answers were classified as wanted (“yes”) or unwanted (“no” or “cannot say”).
- *If they had had depression lasting for at least two weeks during pregnancy.* Responses were “yes” or “no.”
- *If there had been other mental symptoms than depression during pregnancy and what the symptoms were.* Fears were classified into one group, and other mental symptoms (anxiety, panic, obsessions, insomnia, fatigue, tearfulness, sensitivity, psychosomatic symptoms, headache, and loss of appetite) were classified into a second, miscellaneous group. This categorization is based on the fact that fears, especially a fear of childbirth, are specific contributors to PPD [3, 9].
- *If they had had a complicated pregnancy and what the complications were.* The complications were classified as hyperemesis and miscellaneous pregnancy-related complications (pain, diabetes mellitus, anemia, toxemia, preeclampsia, hepatogestosis, pruritus, premature contractions, infection, vaginal bleeding, and small-for-date infant) and infertility treatment. Classification was based on the different nature of these difficulties. For example, hyperemesis is linked with a broad spectrum of mental symptoms and disorders [16], and infertility treatment [3, 17] associates with distress before and during early pregnancy.
- *If they had had a complicated delivery and what the complications were.* The complications were classified as painful labor and miscellaneous complications during delivery (no contractions, lengthy labor, instrumental delivery, excessive bleeding, infection, emergency cesarean section, and fatigue). Categorization was based on the fact that pain is an especially common discomfort during delivery [2, 18]. Mode of delivery (vaginal, elective cesarean section, or emergency cesarean section) was also asked.
- *If the infant has had sicknesses or symptoms and what the sicknesses or symptoms were.* The answers were classified as colic, fetal abnormalities, and others (eating problems, breathing problems, infections, and allergy). Infantile colic is a common burden for mothers [23, 24]. Fetal abnormalities are much rarer and seem to be specific problems for some mothers [3].
- *If they were breastfeeding.* Responses were “yes” or “no.”
- *If they had experienced negative life events during the previous 12 months and what these events were.* Negative life events were classified as a death of a significant other (own or partner's parents, grandparents, siblings, or friends), own or significant other's sickness (husband, parents, or siblings), socioeconomic problems (unemployment, problems at work, housing problems, economic hardship, or academic difficulties), and problems in close relationships (breakdown of relationship or own or parents' divorce). This categorization loosely resembles that used by Ahluwalia et al. [34].

The study protocol was approved by the Ethical Committee of the North Karelian Hospital District Federation of Municipalities. All participants gave their informed consent to participate in this study prior to data collection.

Data analysis was carried out with IBM SPSS Statistics (version 22). To assess the differences between the study groups, also in relation to the course of depression, Pearson's chi-squared test and Fisher's exact test were used for the categorical variables and independent samples *t*-test was used for the continuous variables. If a continuous variable was not normally distributed, a nonparametric Mann–Whitney *U* test was used. A *p* value less than 0.05 denoted statistical significance. The relationships between risk factors and PPD, also in relation to the course of depression, were also investigated using logistic regression. The final age-adjusted logistic regression model included continuous sum variables “pregnancy and delivery issues” (unwanted pregnancy, depression and/or other mental symptoms during pregnancy, complicated pregnancy, and complicated delivery including both vaginal delivery and complicated cesarean section) and “issues relating infant and breastfeeding cessation” (infant's symptoms and illnesses and breastfeeding cessation). Negative life events during the previous 12 months were classified as a binary variable (no = 0; yes = 1).

## 3. Results

The study group consisted of 104 mothers with a major depressive disorder and a control group of 104 nondepressed mothers.

The mean age of the infants was 82.9 days (standard deviation (SD) 33.5) in the depression group and 80.2 days (SD 19.3) in the nondepressed group (*p* = 0.36). Depressed mothers were younger than nondepressed mothers (mean 27.4 years (SD 5.3) versus 29.6 years (SD 4.1), *p* = 0.001). Fifty-three (51.0%) mothers in the depressed group had delivered their first child, as had 48 (46.2%) mothers in the nondepressed group (*p* = 0.49). No difference was found in the mean number of children (mean 1.7 (SD 0.9) versus 1.8 (SD 0.9), *p* = 0.67, resp.). The mean number of gestations was 2.0 (SD 1.3) in the depressed group and 2.1 (SD 1.2) in the nondepressed group (*p* = 0.68). No participant had had a stillbirth. There were no differences between the groups in regard to the number of mothers with previous spontaneous abortion (23 (22.1%) versus 18 (17.3%), *p* = 0.383) or induced abortion (7 (6.7%) versus 9 (8.7%), *p* = 0.60).

Unwanted pregnancy, depression, fears, and other mental symptoms during pregnancy were significantly more common among the depressed than the nondepressed mothers (Table 1). Pregnancy-related problems were as common in both groups (Table 1). Nevertheless, hyperemesis was more common among the depressed than the nondepressed mothers. Complicated delivery—specifically pain—was significantly more common among the depressed than the nondepressed controls (Table 1). There were no differences between groups with regard to method of the delivery (depressed/nondepressed: vaginal delivery 87 (83.7%) versus 90 (86.5%), *p* = 0.559, elective section  7 (6.7%) versus 10 (9.6%), *p* = 0.448, and emergency section  10 (9.6%) versus 4 (3.8%), *p* = 0.097).

Infants suffering from symptoms and illnesses, especially from infantile colic, were more common among depressed than nondepressed mothers (Table 1). Cessation of breastfeeding was more common in depressed mothers.

Negative life events during the previous 12 months were more common among depressed mothers than among the nondepressed controls (Table 1). Specifically, complicated close relationships were more common among depressed mothers.

The number of risk factors was higher in the depressed than in the nondepressed group (Table 2). Eighteen percent (18.2%: 19/104) of the mothers were experiencing their first depression and 81.7% (85/104) recurrent depression. Sixty-nine percent (69.2%: 72/104) of nondepressed mothers had no previous depression. The mean number of risk factors was higher among mothers with first lifetime depression (*n* = 19) than among the never-depressed control group (*n* = 72; mean 2.58 (SD 1.46) versus mean 1.32 (SD 1.11), *p* < 0.001, resp.). Similarly, mothers with recurrent depression (*n* = 85) had more risk factors than their never-depressed counterparts (*n* = 72; mean 2.94 (SD 1.43) versus 1.32 (SD 1.11), *p* < 0.001, resp.). There was no difference between groups with regard to first lifetime (*n* = 19) and recurrent depression (*n* = 85; mean 2.58 (SD 1.46) versus mean 2.94 (SD 1.43), *p* = 0.247, resp.).

Pregnancy and delivery issues, issues relating to the infant and breastfeeding, and negative life events during previous 12 months were associated with PPD according to age-adjusted logistic regression (Table 3). Pregnancy and delivery issues associated with both the risk of first lifetime depression and the risk of recurrent depression when compared to the never-depressed control group. Nevertheless, issues related to the infant and breastfeeding cessation and negative life events associated only with the risk for recurrent depression (Table 3). In a subgroup of mothers who had had at least one previous depressive episode, negative life events during the previous 12 months were a significant risk factor for PPD (OR 2.60, 95% CI 1.00–6.75, and *p* = 0.049). No differences were found in the nondepressed control group between mothers with no history of depression and a history of previous depressions (data not shown).

## 4. Discussion

In general, mental and physical problems during pregnancy or delivery, postpartum problems with the infant and breastfeeding cessation, and negative life events during the previous 12 months were connected to PPD. Mental and physical problems during pregnancy or delivery were associated with both first lifetime depression and recurrent depression during the postpartum period. Furthermore, for recurrent depression, infant/breastfeeding issues and negative life events were also risk factors. The differences in risk factors between first lifetime and recurrent major depressive episodes during the postpartum period represent a novel finding of this study. The risk of recurrent depression is independently associated with more risk factors of lesser severity for the mother's health compared to the risk of first lifetime depression. This means that during the postpartum period a recurrent episode of depression may result from less severe stress exposure than the first lifetime episode, which is in line with the kindling hypothesis [35]. Among mothers who had experienced a previous depressive episode, recent negative life events were of particular importance.

In our study, unwanted pregnancy and an indifferent attitude to pregnancy were connected to depression. An unwanted pregnancy may change life considerably, be a stressful experience with social and economic changes, and further impact on difficulties with motherhood [2, 7, 8, 10].

According to our study, depression and other mental symptoms during pregnancy are important risk factors for PPD. This association has been found in numerous previous studies [2, 6–9]. In our study, fears were emphasized, and these were linked widely to the present life situation, not only to childbirth, as is usually studied [3, 9]. Findings from other studies suggest that pregnancy-related complications are in general potential risk factors for PPD [2, 3, 8, 12, 14, 15], but in our study only hyperemesis as a specific pregnancy complication associated with PPD. Not surprisingly, hyperemesis has been linked with depression, anxiety, and mental health difficulties in other studies [8, 13, 16].

Our study found that complicated delivery, especially pain during delivery, is connected to depression. Other studies [2, 8, 18] have shown mixed findings regarding the link between delivery complications and PPD.

Cesarean section (elective and emergency) was equally as common among depressed and nondepressed mothers, as several previous studies have suggested [2, 8]. Perhaps the pain and other complications during delivery are more remarkable reasons for depression than the method of delivery [2, 8, 18].

Infant-related problems are well-known risk factors for PPD [2, 3, 7, 15, 22]. We found that infant symptoms and sicknesses were connected to depression. According to our study, the main cause of this is infantile colic with uncontrolled crying [23, 24]. Infant crying is associated with the mother's tiredness and may act as a trigger to depression [24]. Our study, like previous studies [2, 11, 25, 26, 28], shows that depressed mothers more commonly have breastfeeding cessations. The effect of breastfeeding cessation on depression may be mediated through poor self-esteem by failed intentions to breastfeed [10, 11, 27]. Alternatively, depressed women may give up breastfeeding more readily [10, 26, 28].

We found that negative life events during the previous 12 months were common among depressed mothers, which is in line with several previous studies [2, 6, 8, 11, 17, 29–31]. Our study highlighted the role of problematic relationships. When first lifetime and recurrent depressive episodes were studied separately, an association was found only with recurrent depressions. This suggests that previous problems in relationships might associate with previous depressions. It may even be that previous problems in relationships and previous depressions have an additive impact on the risk for PPD.

Not surprisingly, the number of risk factors was higher among depressed than nondepressed mothers. Furthermore, the number of risk factors was also higher among mothers with first lifetime depression or recurrent depression during the postpartum period than among nondepressed mothers without a history of depression. A number of stressors have also been connected to PPD in other studies [11, 17, 29–31].

The limitations of this study include the retrospective self-report of previous depressions and experiences of life events, pregnancy, delivery, and the infant. Mothers may downplay or exaggerate their responses according to their beliefs and perceptions, and in the midst of a depressive episode their thoughts may be negative about the self and the environment [36]. It is possible that mothers do not recognize their symptoms and disorders. The threat to internal validity is also the fact that the sampling time was longer in the depressed than in the nondepressed group. Nevertheless, the depressed group and the control group represent the same socioeconomically diverse population. Mothers attended the same obstetric clinic, and the health services were stable during the sampling time. Furthermore, the use of a convenience sampling method limits the generalizability to the broader population. We could only evaluate depressed mothers who accepted an invitation from the primary health care nurses to attend a psychiatric interview. The cross-sectional nature of this study limits the assessment of causality. The small number of cases increased the risk for type II statistical error, so true differences may not have been detected. There may also be intercorrelations between variables. A further limitation is the fact that the psychiatrist (Pirjo Kettunen) conducting the semistructured interviews was not blind to the mothers' depression status. The diagnostic interview constitutes a critical strength of the study. The diagnoses of major depressive disorder in the study group and control group were made by the psychiatrist using the Structured Clinical Interview for DSM-IV Axis I Disorders.

## 5. Conclusions

In conclusion, mental and physical problems during pregnancy or delivery have an impact on both first lifetime and recurrent PPD. Nevertheless, in recurrent depression, adverse experiences with the infant and breastfeeding cessation and negative life events around the time of having a baby also have an important impact. Compared to nondepressed mothers, depressed mothers have more adverse experiences. Of particular importance for consideration are problematic relationships, unwanted pregnancy, depression, fears and other mental symptoms during pregnancy, hyperemesis, pain during delivery, and infantile colic. To be able to prevent and care for PPD in clinical practice, it is necessary to discuss the mother's stress factors with her. Pregnancy planning, mental symptoms during pregnancy, and relationship problems should be considered. It is important to handle hyperemesis and delivery pain well, support women in caring for babies with colic, and provide expert support to women who have difficulties with breastfeeding. If a mother has experienced previous depression, it is especially important to take negative life events into account.

## Figures and Tables

**Table 1:** Adverse experiences during the antenatal and postnatal period in the nondepressed and depressed group.

	Nondepressed group		Depressed group		*p* value^5^
	*N* = 104		*N* = 104		
	*n*	%	*n*	%	
Unwanted pregnancy	6	5.8	21	20.4	0.002
Depression during pregnancy	5	4.8	44	42.3	<0.001
Mental symptoms during pregnancy excl. depression	12	11.5	35	33.7	<0.001
(i) Fears	3	2.9	11	10.6	0.027
(ii) Miscellaneous^1^	9	8.7	27	26.0	0.001

Complicated pregnancy	44	42.3	50	48.1	0.403
(i) Hyperemesis	4	3.8	15	14.4	0.008
(ii) Pregnancy complications^2^	41	39.4	42	40.4	0.887
(iii) Infertility treatment	4	3.8	1	1.0	0.366^6^

Complicated delivery	31	29.8	46	44.7	0.031
(i) Pain	7	6.7	20	19.2	0.007
(ii) Miscellaneous^3^	24	23.1	27	26	0.629

Infant's symptoms and illnesses	14	13.5	32	30.8	0.003
(i) Infantile colic	6	5.8	17	16.3	0.015
(ii) Fetal abnormalities	4	3.8	5	4.8	1.000^6^
(iii) Miscellaneous^4^	4	3.8	10	9.6	0.097
Breastfeeding cessation	18	17.3	40	38.5	0.001

Negative life events	27	26.0	47	45.2	0.004
(i) Death of significant others	12	11.5	13	12.5	0.831
(ii) Sickness (own or significant others)	8	7.7	16	15.4	0.083
(iii) Relationship problems	5	4.8	17	16.3	0.007
(iv) Socioeconomic problems	4	3.8	8	7.7	0.234

^1^Anxiety, panic, obsessions, insomnia, fatigue, tearfulness, sensitivity, psychosomatic symptoms, headache, and loss of appetite. ^2^Pain, diabetes mellitus, anemia, toxemia, preeclampsia, hepatogestosis, pruritus, premature contractions, infection, vaginal bleeding, and small-for-date infant. ^3^No contractions, lengthy labor, instrumental delivery, excessive bleeding, infection, emergency section, and fatigue. ^4^Eating problems, breathing problems, infections, and allergy. ^5^Pearson's chi-square test. ^6^Fisher's exact test.

**Table 2:** Number of risk factors in the nondepressed and depressed group.

Number of risk factors^1^	Nondepressed group		Depressed group	
	*N* = 104		*N* = 104	
	*n*	%	*n*	%
0	22	21.2	4	3.8
1	37	35.6	15	14.4
2	28	26.9	23	22.1
3	7	6.7	26	25.0
4	8	7.7	25	24.0
5	2	1.9	7	6.7
6	0	0	3	2.9
7	0	0	1	1.0

	Mean^2^ = 1.50	SD = 1.23	Mean^2^ = 2.87	SD = 1.44

^1^Unwanted pregnancy, depression and/or other mental symptoms during pregnancy, complicated pregnancy, complicated delivery, infant's symptoms and illnesses, breastfeeding cessation, and negative life events during the previous 12 months. ^2^Mann–Whitney *U* test: *p* < 0.001. SD = standard deviation.

**Table 3:** Age-adjusted risk for having postpartum depression according to multivariate logistic regressions in relation to the course of depression.

	Depressed (*n* = 104) versus nondepressed mothers (*n* = 104)			Mothers with first lifetime depression (*n* = 19) versus never-depressed controls (*n* = 72)			Mothers with recurrent depression (*n* = 85) versus never-depressed controls (*n* = 72)		
	OR^1^	95% CI^2^	*p* value	OR^1^	95% CI^2^	*p* value	OR^1^	95% CI^2^	*p* value
Pregnancy and delivery issues (*n* ^3^ = 4)	2.31	1.59–3.35	<0.001	2.87	1.33–6.17	0.007	2.88	1.81–4.59	<0.001
Issues relating infant and breastfeeding cessation (*n* ^4^ = 2)	1.86	1.11–3.09	0.018	2.27	0.96–5.35	0.062	2.17	1.14–4.13	0.019
Negative life events (no versus yes)	2.16	1.12–4.16	0.021	1.00	0.28–3.58	1.000	2.51	1.15–5.50	0.021

^1^OR = odds ratio. ^2^95% CI = 95% confidence interval. ^3^Unwanted pregnancy, depression and/or other mental symptoms during pregnancy, complicated pregnancy, and complicated delivery. ^4^Infant's symptoms and illnesses and breastfeeding cessation.
